# Editorial Comment: De novo urinary incontinence after pelvic organ prolapse surgery-a national database study

**DOI:** 10.1590/S1677-5538.IBJU.2021.02.05

**Published:** 2021-02-03

**Authors:** Cássio L. Z. Riccetto

**Affiliations:** 1 Universidade Estadual de Campinas Faculdade de Ciências Médicas Divisão de Urologia Feminina Campinas SP Brasil Divisão de Urologia Feminina - Faculdade de Ciências Médicas da Universidade Estadual de Campinas - UNICAMP, Campinas, SP, Brasil

Yasmine Khayyami ^1^, Marlene Elmelund ^2^, Gunnar Lose ^2^, Niels Klarskov ^2^

## COMMENT

In this study, the authors concluded that the risk of de novo incontinence after surgery to treat pelvic organ prolapse (POP) was 15%. This data was collected from the Danish National Registry, which included 1.198 patients operated between 2013 and 2016. Elevated body mass indexes (BMI) strongly correlated with the risk de novo incontinence (12% for women with BMI <25; 16% for women with BMI 25 - <30; and 23% for those with BMI ≥ 30). Meanwhile, no correlations was found between the risk of de novo incontinene and age at surgery, vaginal compartment involvement or POP staging. To define continence, the authors used the International Consultation on Incontinence Questionnaire-Urinary Incontinence-short form (ICIQ-UI-SF) according to which a woman was urinary continent if her total score was zero and she answered ‘never’ to ‘When does urine leak?’

The rationale regarding de novo urinary incontinence after POP surgical treatment is that the procedure could lead to an imbalance of pelvic floor dynamics, preventing the force vectors produced by the pelvic floor muscles and fascia to keep the urethral closure in response to changes in abdominal pressure. De novo incontinence represents a condition with a strong psychological impact on patients, and differs from occult urinary incontinence, which can be demonstrated preoperatively through POP reduction maneuvers. In this sense, in a recent study from the Spanish Pelvic Floor Dysfunction in Women Investigation Group, occult incontinence was detected after POP reduction in 59% of patients who underwent urodynamics (1). Previous studies have tried to determine the predictors of de novo incontinence, such as patient age and comorbidities such as diabetes mellitus (2, 3). The conclusion from the present study mainly highlights the relevance of BMI for the pre-operative counseling of POP patients.
